# Non-contact measurement of facial surface vibration patterns during singing by scanning laser Doppler vibrometer

**DOI:** 10.3389/fpsyg.2015.01682

**Published:** 2015-11-03

**Authors:** Tatsuya Kitamura, Keisuke Ohtani

**Affiliations:** ^1^Faculty of Intelligence and Informatics, Konan UniversityKobe, Japan; ^2^Faculty of Education, Nara University of EducationNara, Japan

**Keywords:** scanning laser Doppler vibrometer, vibration velocity, resonance, vocal register, pitch frequency

## Abstract

This paper presents a method of measuring the vibration patterns on facial surfaces by using a scanning laser Doppler vibrometer (LDV). The surfaces of the face, neck, and body vibrate during phonation and, according to Titze (2001), these vibrations occur when aerodynamic energy is efficiently converted into acoustic energy at the glottis. A vocalist's vibration velocity patterns may therefore indicate his or her phonatory status or singing skills. LDVs enable laser-based non-contact measurement of the vibration velocity and displacement of a certain point on a vibrating object, and scanning LDVs permit multipoint measurements. The benefits of scanning LDVs originate from the facts that they do not affect the vibrations of measured objects and that they can rapidly measure the vibration patterns across planes. A case study is presented herein to demonstrate the method of measuring vibration velocity patterns with a scanning LDV. The objective of the experiment was to measure the vibration velocity differences between the modal and falsetto registers while three professional soprano singers sang sustained vowels at four pitch frequencies. The results suggest that there is a possibility that pitch frequency are correlated with vibration velocity. However, further investigations are necessary to clarify the relationships between vibration velocity patterns and phonation status and singing skills.

## 1. Introduction

Phonation induces vibration not only in the air column of the vocal tract, but also in body tissues. Body tissue vibrations could cause speakers or singers to experience sympathetic or vibratory sensations. Especially while producing a resonant voice, a vocalist experiences voice self-generation inside of his or her head and on the surface of his or her face. Titze (2001) gave a theoretical explanation of the basis of such sensations, specifically, that they occur when aerodynamic energy is efficiently converted into acoustic energy at the glottis. Singers have utilized such sensations to evaluate their own singing statuses. Miller (2004), a vocal coach, affirmed that “When the spectral balance is complete, a singer is aware of sensations in bony structures of the head that are quite different from those of imbalanced phonation.” Therefore, if the vibrations related to these sensations could be observed from outside of the body, the data could likely be used to indicate vocalists' phonatory states or singing skills.

Phonatory body vibrations have been measured, and the relationships between phonation and these vibrations have been explored for the past 50 years (Sundberg, 1992). In the early stages of investigation, contact-type vibrometers were utilized. Kirikae et al. (1964) measured the vibrations of more than 40 points on subjects' bodies by using a bone conduction receiver. They presented the detailed vibratory amplitude distributions obtained while the subjects spoke Japanese vowels. Sundberg (1997) observed participants' chest wall vibrations during singing by using accelerometers and found that the amplitudes of the fundamental frequency components of the participants' voices were correlated with their chest wall vibration amplitudes. Suzuki et al. (1991) employed small and light acceleration pickups and measured the vibration accelerations of the nasal, neck, and cheek walls during speech production. Lamarche and Ternstöm (2008) measured the skin acceleration level near the glottis and statistically evaluated whether such measurements could be used in phonetgrams. However, it is undeniable that sensors used in contact with measured objects affect their vibrations, although light and small acceleration pickups have been available recently.

On the other hand, non-contact-type vibrometers enable measurement of the vibrations of object surfaces without affecting their vibrations. Pawluczyk et al. (1982) and Pawluczyk and Kraska (1985) developed a laser-based non-contact method that can be used to obtain objects' vibration interferograms and measured the nodal patterns on singer's facial and neck surfaces. Toyoda and Fujinami (2009) employed a fiber-optic Doppler sensor to measure the vibrations at nine different locations on vocalist's body during singing. They found that the vibration amplitude near the fundamental frequency of the singer's voice increased when she expanded her laryngeal and pharyngeal cavities during voicing. Avargel and Cohen (2011) attempted to obtain speech information from neck surface vibrations by using a single-point laser Doppler vibrometer (LDV).

Recently, Kitamura (2012) was the first to employ another laser-based non-contact method, a scanning LDV, to measure the vibration velocity patterns of participants' facial surfaces during speaking. In the experiment, male participant lay supine on the floor, and the vibration velocities of about 50 points on his face were measured while he produced a sustained vowel /a/ and a nasal consonant /N/. The results demonstrated that the method could clearly measure vibration velocity amplitude differences on the lateral nasal walls. Kitamura et al. (2013) next demonstrated that the scanning LDV could measure facial vibration patterns while participants sang in upright body positions. The vibration patterns showed different trends for front and back vowels and for modal and falsetto voices; however, the measurement were only performed once for each condition, and the results were not analyzed statistically. Kitamura (2014) also assessed the reproducibility of measurements obtained using the scanning LDV by analyzing the vibration velocity variations at about 60 points on singers' faces. The results showed that the root-mean-square errors of the facial surface vibration velocities were less than 4.0 dB.

In this paper, we propose a method of measuring facial surface vibration patterns during phonation by using the scanning LDV and discuss its technical merits and demerits, based on the previous studies (Kitamura, 2012, 2014; Kitamura et al., 2013). In addition, we present example measurements of facial surface vibration patterns during sustained singing and examine the relationships between the measured vibration velocity patterns and pitch frequency and vocal registers.

## 2. Methods

In this section, we propose a method of obtaining facial surface vibration patterns by using a scanning LDV. The method is applicable to measurements not only of facial vibrations but also of those on other parts of the body.

### 2.1. Laser doppler vibrometer

An LDV is an optical transducer that applies a laser beam to a vibrating surface and measures the frequency shift of the laser beam reflected from the surface. It can determine the vibration velocity and displacement at any point on the surface based on the Doppler effect. A scanning LDV can automatically scan and probe multiple pre-selected points on an object's surface and obtain their vibration patterns, while a single-point LDV can only measure vibrations at a single point.

**Figure 1:**
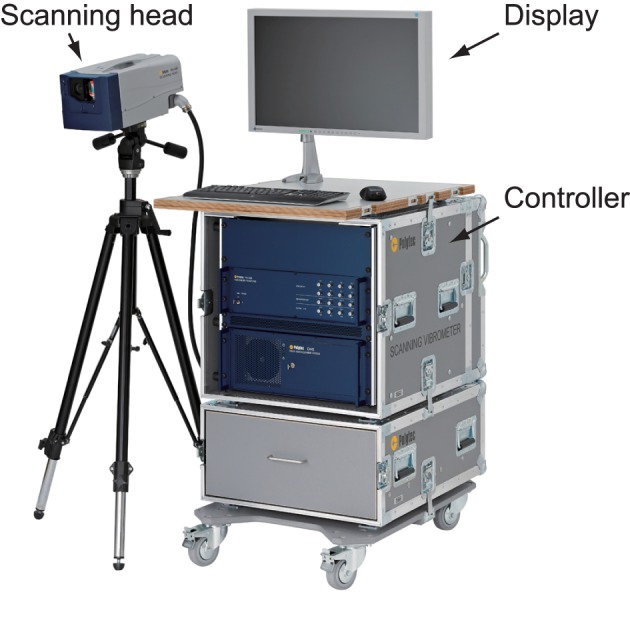
**Scanning laser Doppler vibrometer, Polytec PSV-500**.

In this study, we employed a scanning LDV, Polytec PSV-500, to measure facial surface vibration velocities. The system consisted of a scanning head that applied and received the laser beam, a controller, and software for acquiring and displaying the measured data. Figure 1 shows the hardware of the system. The diameter of the laser beam, defined as the width at which the intensity of the laser beam is 13% of the value at its center was less than 100 μm.

### 2.2. Advantages and disadvantages of LDVs

The biggest advantage of LDVs is that they enable contactless measurement of the vibration velocity and displacement of an arbitrary point on a vibrating surface. LDVs therefore do not affect objects' vibrations, while an acceleration pickups, which are the transducers commonly used to measure vibrational acceleration, could affect objects' vibrations. Scanning LDVs can also quickly control their laser beam irradiation angles and automatically scan multiple points. They enable rapid measurement of vibration patterns across large surface areas and, consequently, can reduce the burden on participants.

On the other hand, the current LDVs cannot account for measurement point misalignment during data acquisition and cannot automatically track points. That is, to measure the exact vibration velocity and displacement of a measured object, it should not be moved during measurement. This characteristic could become problematic during surface vibration measurement of a living subject.

### 2.3. Head stabilization

While measuring the facial surface vibrations of participants during singing using LDVs, it is indispensable to keep their heads immobile since such devices are sensitive to subject movements, as mentioned above. We investigated the following three head stabilization methods.

In the first method, each participant lay supine on the floor with his or her head stabilized by wooden blocks (Kitamura, 2012). This method could keep the participants' heads highly stable; however, it is possible that the vibration patterns in the supine posture are different from those in an upright position because body posture could change the shape of the vocal tract during speech production (Kitamura et al., 2005). Also, this method might have caused head vibrations to be absorbed because the backs of the participants' heads were in contact with the floor during measurement.

In the second method, each participant sat on a high-backed chair with his or her head was held on each side by a steel pole covered with polyurethane foam and clamped to the back of the chair. The participant's neck was also stabilized by a vacuum cushion (Kitamura et al., 2013). In the third method, each participant sat on a chair and pushed his or her head against a horizontal bar and also clutched vertical poles to stabilize his or her head and body. This apparatus is known as a head support stand (SR Research, Head Support), without a chin-receiving stand (Kitamura, 2014). The last two methods allowed the participants to be upright, a more natural body position for singing; however, it was rather difficult for them to keep their heads immobile while singing.

Even if participants' heads could be kept in fixed positions, the surfaces of their faces could still move; for example, the facial surfaces could move during speaking and singing as a result of emotional expression (Livingstone et al., 2009, 2015), and the eyebrows could move in tandem with the pitch frequency of the voice (Guaïtella et al., 2009). If an object-tracking vibrometer such as a VibroTracker (Miyashita et al., 2013) could be employed, it might be possible to perform measurements even while participants' heads and faces move.

### 2.4. Reproducibility of measurements

Kitamura (2014) assessed the reproducibility of facial surface vibration velocity measurements obtained during singing by using the scanning LDV. In the experiment, each of three trained singers sat upright in a chair and fixed his or her head and body using the head support apparatus mentioned in the description of the third head stabilization method in the previous section. Each participant sang a Japanese vowel /a/ continuously, and the vibration velocities of 59 points on his or her facial surface were measured. Three sets of measurements were performed for each participant, and the resulting RMS errors were less than 4.0 dB for each of the measurement points.

### 2.5. Laser emission safety

The scanning LDV, Polytec PSV-500, employs a class-II helium-neon laser (the wavelength is 633 nm), which could cause injury if a person were to gaze into the laser beam. To protect the participants' eyes from the laser emission during the experiments, we first asked them to wear swimming goggles covered by lightproof fabric tape; however, without vision, it was difficult for the participants to stabilize their heads and unsteadiness of a measured object directly decreases the resulting LDV measurement accuracy. Thus, in the present study, we covered the swimming goggles with 3-mm-thick laser safety filters (Riken Optech, RLF-He), which could filter out the helium-neon laser beam but did not deprive the participants' vision.

## 3. Measurement method example

This section presents descriptions of the method and results of facial surface vibration pattern measurements performed using the scanning LDV. Female singing voices are generally divided into three vocal registers, the modal, mixed, and falsetto registers, based on their tonal ranges. The modal and falsetto registers are the lowest and highest registers, respectively. While singing in the falsetto register or head register, a singer experiences self-generated voice functioning at the top of or inside of the head (Warrack and West, 1992). If these sensations result from the vibrations of the bony structures and the tissues of the head, the skin surface of the head could vibrate during singing in the falsetto register. This example was thus employed to preliminarily investigate the vibration pattern differences on singers' foreheads while they sang in their modal and falsetto registers.

### 3.1. Participants

Three soprano singers, A, B, and C (in their 20, 30, and 40 s, respectively), who had performed professionally in operas and concerts, participated in the experiments. Their singing histories are listed in Table 1. The mean and standard deviation of their ages were 35 and 7, respectively. All of the participants were native Japanese speakers. Since it was necessary for the participants to sing in a supine posture in the experiments, we found singers who reported that they could sing naturally in various body postures. They were paid for their participation in the experiment.

**Table 1:** **Singing experience of participants (years)**.

	**Singer A**	**Singer B**	**Singer C**
Number of years of	12	16	30
singing experience			
Number of years of	12	16	30
private singing lessons			
Number of years of	4	28	35
group singing experience			

### 3.2. Tasks

Each of the participants sang the Japanese vowels /a/ and /i/, which are back and front vowels, respectively, in a supine posture. The singers produced these vowel sounds in their modal and falsetto voices for the four pitch frequencies listed in Table 2. Since the participants claimed that continuous singing in the modal voice could injure their vocal cords, we asked them to sing in the modal voice only for the vowel /a/. The singing voices are hereinafter referred to as PF1, PF2, PF3, and PF4 in order of increasing pitch frequency, where PF1 corresponds to the modal voice and the others indicate the falsetto voices. Prior to each measurement, one of the experimenters played a note on a piano so that the participants could confirm the pitch that she would be asked to produce next. The participants sang at comfortable volumes, i.e., we did not control the loudness of their singing voices.

**Table 2:** **Pitch frequencies of participants' singing voices**.

**Singer**	**ID**	**Pitch frequency**
A	PF1	A3 (220 Hz)
	PF2	F5 (698 Hz)
	PF3	G5 (784 Hz)
	PF4	A5 (880 Hz)
B	PF1	C4 (261 Hz)
	PF2	F5 (698 Hz)
	PF3	G5 (784 Hz)
	PF4	A5 (880 Hz)
C	PF1	A3 (220 Hz)
	PF2	F5 (698 Hz)
	PF3	A5 (880 Hz)
	PF4	C6 (1,047 Hz)

### 3.3. Procedures

**Figure 2:**
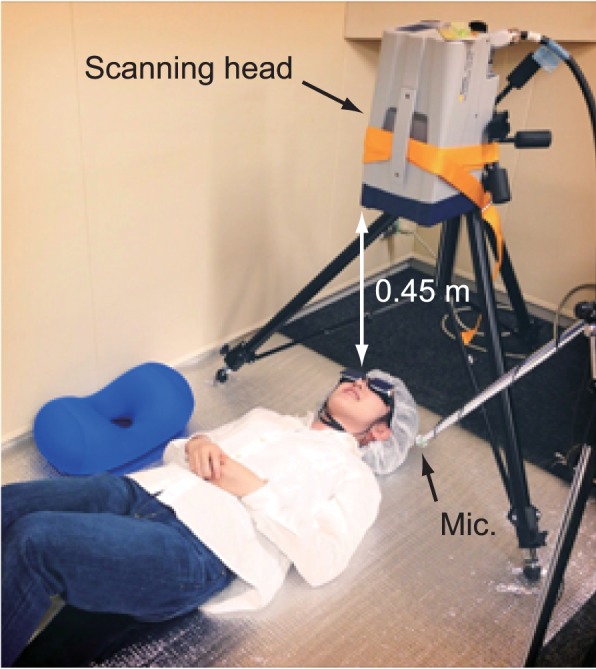
**Configuration of measurement instruments and participant**.

The measurements were conducted in a soundproof room. Each participant first warmed up according to her personal routine and then was asked to lay supine on a mat, to wear a non-woven fabric hat to keep her hair away from her forehead, and to wear the laser safety goggles. A towel was placed under the back of each participant's neck to stabilize her head. The scanning head of the vibrometer was mounted on a tripod to set the optical axis perpendicular to the floor. Each participant's head was positioned directly beneath the scanning head approximately 0.45 m from it. The arrangement of the scanning head and the participant is shown in Figure 2.

**Figure 3:**
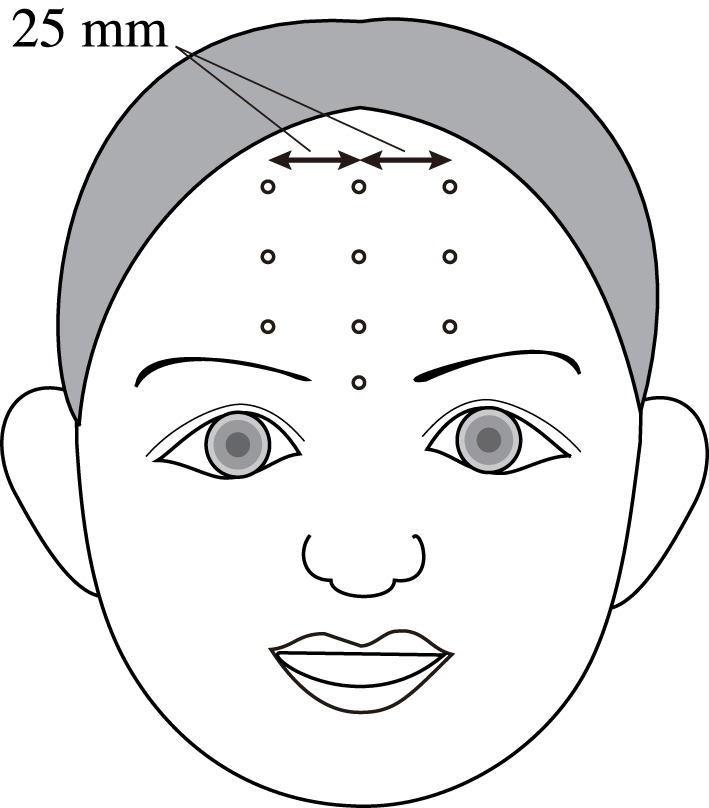
**Ten points used for vibration velocity measurements**.

In the present study, the vibration velocities were measured at 10 points on each participant's forehead. The measured points included four points on the mid-sagittal plane and three points on both the right and left sagittal planes, which were approximately 25 mm from the mid-sagittal plane, as shown in Figure 3. The vertical distances between the measured points were even intervals that were determined separately for each participant and depended on forehead height. Adhesive markers were placed on each participant's forehead to mark the three sagittal planes, and the measured points were then set manually by the control software of the vibrometer while the participant held her face as if singing. If the measured points were misaligned due to head movement during the experiment, the points were reset.

The vibration velocities, whose units are m/s in dB, at each of the measured points were acquired as frequency-domain data at a sampling frequency of 7813 Hz. The sampling frequency was determined based on the fact that the vibration velocity peak was attenuated considerably over approximately 3 kHz. A high-pass filter with a cut-off frequency of 10 Hz removed the background vibration of the building from the data. The data were remeasured automatically if valid vibration velocity data could not be acquired for reasons such as insufficient reflection of the laser.

The vibration velocity measurement at each point was triggered when the participant's singing voice was recorded by a microphone (Sony, ECM-77B) and an amplifier (Edirol, UA-5). The microphone was approximately 0.5 m from the participant's mouth in each case. Data acquisition for each point required 0.256 s, depending on the sampling frequency and the FFT length (800 points in the present study) for the vibration velocity. Consequently, including scanning and remeasurement, approximately 6–10 s was needed to measure the vibration velocities of the 10 points. The measurements for each participant could be finished within approximately one breath.

The equivalent noise level (LAeq) was measured concurrently with the vibration velocities and singing voice. LAeq is the A-weighted sound pressure level in a period of time and is measured in dB, that is, it is the sound pressure level corrected according to the equivalent loudness contours representing the loudness perceived by the human hearing system. The microphone of a sound level meter (RION, NL-20) was positioned approximately 1 m from each participant's mouth, and the maximum value of the digitally represented equivalent noise level was recorded.

These measurements were conducted three times for each pitch frequency and were blocked according to pitch frequency. Thus, the design of this experiment was a vowel (2 levels) × pitch frequency (4 levels) × repetition (3 levels), following the within-subjects design, and the measurements were blocked according to pitch frequency. The entire measurement process, including rest intervals, took approximately 30–40 min. Written informed consent was obtained from each participant prior to the measurements, and the experimental protocol was approved by the ethical and safety committees of Konan University.

### 3.4. Data analysis

In this experiment, the vibration velocities were acquired in the frequency domain, that is, the amplitude of the log spectrum of the vibration velocity at each discrete frequency up to the Nyquist frequency was stored in a PC. The amplitudes of the vibration velocities were obtained in units of dB, and 1 m/s was normalized to 0 dB. The normalization was performed automatically by the control software of the vibrometer. The Nyquist frequency, which is defined as one-half of the sampling frequency, was 3906.5 Hz in this experiment. For further analysis, the vibration velocity amplitude in a 50 Hz band around each pitch frequency was calculated by employing the band-pass filtering function of the control software. The data measured at the 10 points were averaged, and then mean and standard deviation were calculated for each note and vowel.

## 4. Results and discussion

**Figure 4:**
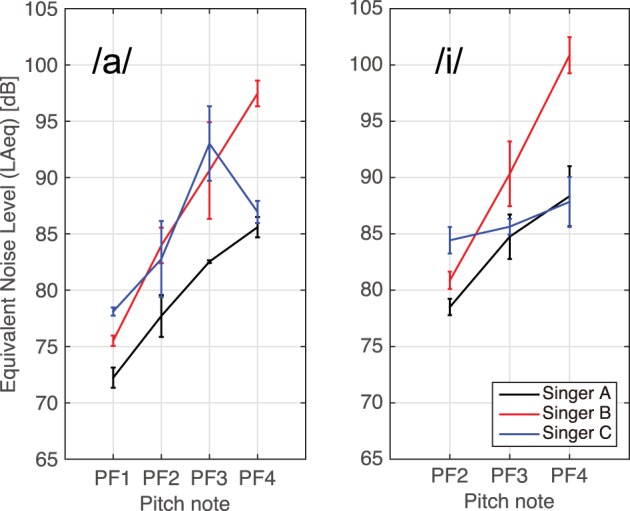
**Equivalent noise levels of singing voices for vowels (left) /a/ and (right) /i/**. Error bars represent standard deviations.

The mean LAeq values of the singing voices are depicted in Figure 4. As shown, LAeq increases with pitch frequency, as is well-known (Baken and Orlikoff, 2000), except in the case of the vowel /a/ for Singer C. The variation between the LAeq values of the different vowels is less than 4 dB for each participant, except for the LAeq for PF3 of Singer C, which varies by 7.4 dB from Singer C's other LAeq values.

**Figure 5:**
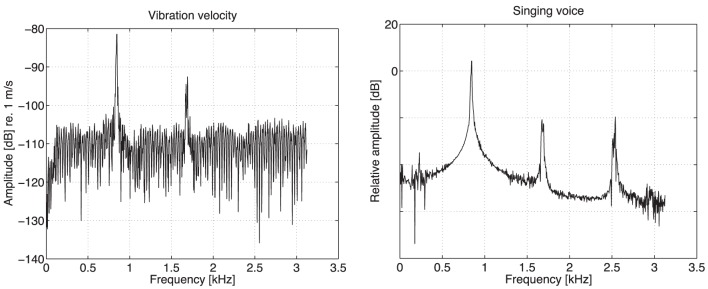
**Log spectra of forehead vibration velocity measured by scanning LDV at one point (left) and singing voice measured by microphone (right) for vowel /a/ of Singer A**. Vibration velocity units are m/s in dB.

Figure 5 shows representative vibration velocity and singing voice spectra, which were measured at the lowest mid-sagittal point for the vowel /a/ of Singer A. The frequencies of the spectral peaks of the vibratory and acoustically measured spectra are in good agreement, whereas the spectral peaks of the vibration velocity disappear 2 kHz.

**Figure 6:**
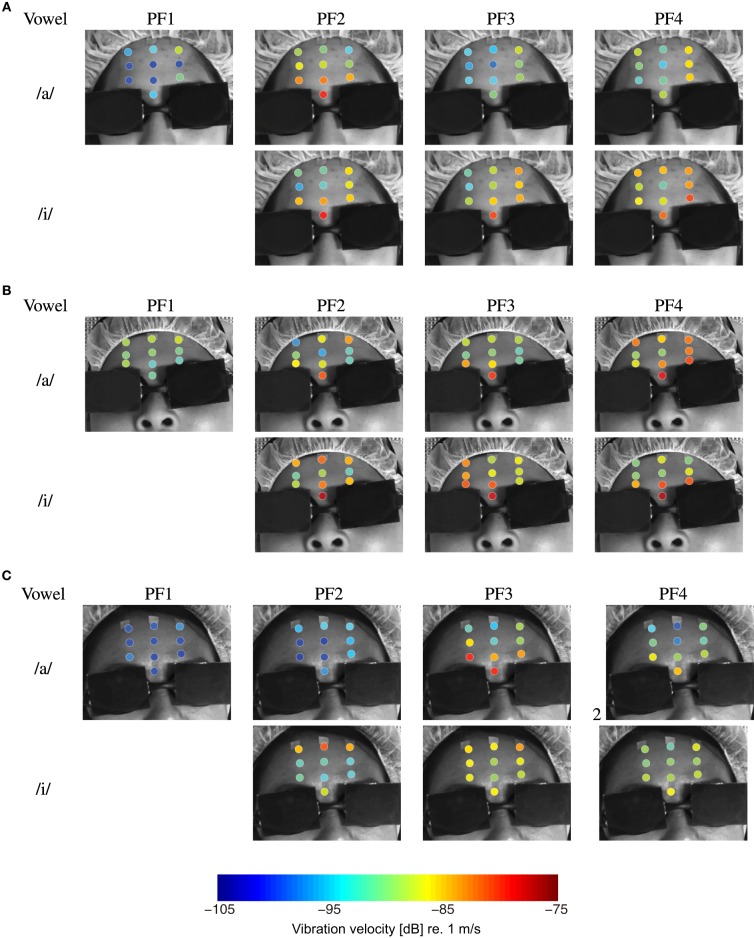
**Forehead surface vibration velocities of three singers while singing Japanese vowels /a/ and /i/ for four pitch frequencies, PF1, PF2, PF3, and PF4**. Velocities are given in m/s (dB), where 0 dB is equivalent to 1 m/s. Singer **(A)**, Singer **(B)**, and Singer **(C)**.

Figure 6 presents the mean vibration velocities calculated from the three measurements taken at each point for the three singers. The mean values in dB are represented on a 64-step color scale, where the vibration velocity decreases as the color becomes colder. The black rectangular plates on the eyes are the laser safety filters. The results reveal that the vibration velocity varies with the location on the forehead and the pitch frequency, and the vibration patterns differ between participants. However, the results may have been affected by vibration velocity variation throughout the duration of singing because the time lags between measurements taken at different points were ignored in this study.

Table 3 lists the means and the standard deviations of the vibration velocities at the 10 measured points as functions of pitch frequency. The relatively large standard deviations indicate that the vibration velocities varied widely for each singer. To evaluate the effects due to pitch frequency and variations between participants, a Two-Way ANOVA was performed on the data with repeated measures for every vowel. For the vowel /i/, only two-thirds of the data for each pitch frequency were analyzed since there were some missing data. Both the pitch frequency and participant factors approached significance: Pitch frequency $F_{\left(3,\;24\right)}=2.77,p=0.06,\eta^{2}=0.13$; Participant $F_{\left(2,\;24\right)}=3.17,p=0.06,\eta^{2}=0.10$, for the vowel /a/. The two-way interaction between pitch frequency and participant was significant, $F_{\left(6,24\right)}=4.45,p<0.01,\eta^{2}=0.41$. For the vowel /i/, the pitch frequency factor was insignificant [$F_{\left(2,16\right)}=0.27,p=0.77,\eta^{2}=0.02$], while the participant factor was significant [$F_{\left(2,\;16\right)}=7.96,p<0.01,\eta^{2}=0.48$], and there was no interaction between these factors [$F_{\left(4,\;16\right)}=0.21,p=0.93,\eta^{2}=0.03$]. Thus, for the participants in this experiment, the results suggest that the pitch frequency contributed to the vibration velocity amplitude for the vowel /a/, and there were significant differences between the singers for the vowels, however further study will be needed confirm this relationship.

**Table 3:** **Means and standard deviations (S.D.s) of forehead vibration velocity while singing Japanese vowels /a/ and /i/**.

**Singer**	**Pitch note**	**/a/**		**/i/**	
		**Mean**	**S.D**.	**Mean**	**S.D**.
A	PF1	−95.5	2.5	–	–
	PF2	−84.6	5.6	−84.7^*^	4.1
	PF3	−91.7	4.3	−85.5	1.2
	PF4	−88.1	2.8	−84.2	3.6
B	PF1	−89.9	0.2	–	–
	PF2	−86.9	3.5	−85.5^*^	1.2
	PF3	−86.2	6.9	−82.9	0.8
	PF4	−82.7	4.3	−83.1	3.6
C	PF1	−99.4	2.5	–	–
	PF2	−96.9	2.2	−87.1	1.5
	PF3	−83.9	3.2	−86.4	3.4
	PF4	−89.5	0.5	−88.8	3.6

*Values are given in m/s (dB), where 0 dB is equivalent to 1 m/s*.

* *One of three measurements was unavailable*.

We also investigated the possibility of correlations between the mean vibration velocity and LAeq; however, no or weak correlations were found between them [Singer A /a/, *r*_(10)_ = −0.24, *p* = 0.46, /i/, *r*_(6)_ = −0.12, *p* = 0.77; Singer B /a/, *r*_(10)_ = 0.45, *p* = 0.14, /i/, *r*_(6)_ = −0.05, *p* = 0.91; Singer C /a/, *r*_(10)_ = 0.60, *p* = 0.04, /i/, *r*_(7)_ = −0.46, *p* = 0.21]. Lamarche and Ternstöm (2008) examined the skin acceleration levels (SALs) measured near participants' vocal folds while singing and reported that the SALs were positively correlated with the musical dynamic level. Svec et al. (2005) reported similar results for the SALs obtained while speaking. It is possible that the vibrations measured near the glottis depended directly on the phonatory effort, but the vibration on the forehead did not.

The results of this experiment suggest that the pitch frequency is correlated only for the vowel /a/ and the singing voice amplitude is not correlated with the vibration velocity on the forehead. Titze (2001) confirmed that when energy is efficiently converted from aerodynamic energy into acoustic energy at the glottis, the vibrations are distributed all over the head, neck, and thorax. Thus, if the energy conversion efficiency at the glottis could be measured by direct or indirect methods, it might be possible to determine the relationship between the energy conversion efficiency and the vibration velocities on the facial surfaces and to experimentally verify Titze's theoretical explanation.

In this experiment, we set the data acquisition time for each point to 256 ms, and, consequently, the total data acquisition time for the 10 points was approximately 6–10 s. As a result, the measurements for each participant could be performed within approximately one breath, resulting in only a minor burden for the participants, representing one advantage of using the scanning LDV. If we had tried to obtain the same data with a single-point LDV, it would have been necessary to adjust the position and angle of its head to measure each different point, requiring significantly more time. On the other hand, we were compelled to measure the skin vibration velocities in rather short times; therefore, the properties of sustained vibrations could not be examined. Thus, there is undoubtedly a trade-off between the burden on participants and the quality of the measured data.

## 5. Conclusions

This article presented a method of measuring the vibration patterns on the surfaces of the face and body during phonation using an optical transducer. Acceleration pickups are generally used to measure vibrations, but they inevitably affect the vibrations of measured objects. LDVs, such as that used in this study, enable non-contact vibration velocity and displacement measurements at multiple measurement across vibrating surface areas based on the Doppler effect. Such systems can measure vibration patterns without affecting the vibration of measured surfaces distinguishing LDVs from other vibration transducers.

An example facial surface vibration measurement method and the corresponding results obtained during singing with a scanning LDV were also presented in this paper. In the experiment, we measured the vibration velocities at 10 points on the foreheads of expert singers while they sang in their modal and falsetto registers. The results exhibited a possibility of significant correlation between pitch frequency and vibration velocity amplitude for the vowel /a/. However, only three singers participated in the experiment, and further measurements are therefore required to conclusively determine the relationships between forehead vibration velocity patterns and pitch frequency and vocal register.

### Conflict of interest statement

The authors declare that the research was conducted in the absence of any commercial or financial relationships that could be construed as a potential conflict of interest.
